# Next-Generation Neurotechnologies Inspired by Motor Primitive Model for Restoring Human Natural Movement

**DOI:** 10.34133/research.0942

**Published:** 2025-11-12

**Authors:** Ze-Jian Chen, Xiao-Lin Huang, Nan Xia, Ming-Hui Gu, Jiang Xu, Min Lu, Hong Chen, Cai-Hua Xiong, Yong Chen

**Affiliations:** ^1^ Department of Rehabilitation Medicine, Tongji Hospital, Tongji Medical College, Huazhong University of Science and Technology, Wuhan 430030, China.; ^2^ World Health Organization Cooperative Training and Research Center in Rehabilitation, Wuhan 430030, China.; ^3^ Institute of Medical Equipment Science and Engineering, Huazhong University of Science and Technology, Wuhan 430374, China.

## Abstract

Advances in neuroengineering and artificial intelligence are transforming the landscape of motor rehabilitation, aiming to restore human movement as natural as possible. In recent decades, more advanced interventions are increasingly achievable via hybrid robotic systems, neuroprosthetics, and brain–computer interfaces. However, a fundamental gap of these neurotechnologies remains in modeling the complexity of neuromotor control, particularly how the central nervous system coordinates high-dimensional motor outputs in naturalistic behaviors. Rooted in theoretical neuroscience, the motor primitive (MP) model proposes an adaptable framework to deconstruct and reproduce motor tasks through low-dimensional modules. Interestingly, recent studies have indicated that the MP model may reform current-generation neurotechnologies by digitally shaping the course of human–machine interaction. In this narrative review, we will critically examine conventional target settings and identify their limitations in guiding biomimetic control in neurotechnologies. We then introduce the MP model with its machine learning and physiological scaffolds for better understanding and replicating human natural movement. Finally, we will present its potential in facilitating the next-generation neurotechnologies across kinematic, muscular, and neural domains. By modeling motor control in human and neuroengineering, we believe that the MP-inspired paradigms can initiate a new era of intelligent, patient-specific, and naturalistic motor restoration for various neurological and traumatic diseases.

## Introduction

The generation of coordinated movements is fundamental to motor neuroscience, supporting essential functions such as mobility, hand dexterity, and self-care in humans [1,2]. However, various neurological and traumatic conditions, such as stroke, spinal cord injury (SCI), and amputation, can impair volitional motor function and activities of daily living over human lifespan [3]. The resulting manifestations in patients can be persistent and heterogenous, including weakness, spasticity, tremors, coordination disorders, and even limb loss [4–6]. Consequently, restoring natural human movement remains a substantial challenge for multidisciplinary researchers worldwide [7,8].

In recent decades, various cyborg and hybrid systems have emerged to aid motor rehabilitation, including robotics, neuroprosthetics, and brain–computer interfaces (BCIs) [9–11]. A key feature of these neurotechnologies is their ability to collect vast amounts of data from biosensors to deliver practice or stimuli in a closed-loop scheme [12]. However, clinical trials have often yielded suboptimal results, in large part due to insufficient understanding of how the brain orchestrates the high-dimensional musculoskeletal system to produce normal motor outputs [8]. Modern recording techniques now capture multi-modal data simultaneously with the neural dynamics of human movements. Studies at the neuron population level also allows describing how the brain generates coordinated behavior. As a result, latent representations of human body from different physiological perspectives can be quantified and transform these conventional neurotherapeutics [13]. In this review, we specifically proposed the concept of next-generation neurotechnologies (NGNTs) distinguished from current-generation approaches, which fail to spatiotemporally mimic the characteristics of human motor execution.

To address this critical gap, a paradigm shift is needed in human–machine interaction (HMI) settings to realize the importance of sculpting physiological movement patterns in a spatiotemporal manner. Accordingly, the major challenge lies in the limited understanding of the neuromotor control process. It is pivotal to interpret biophysical data within a solid neuroscience framework for reproducing natural motor sequences [14]. At present, traditional technologies have prominent mismatch between HMI targets and physiological biomarkers that reflect neural control of complex behaviors [15]. For instance, neuroimaging can aid in characterizing changes related to motor dysfunctions [16–18]. The identified features are statistically useful for precision medicine, such as patient stratification, risk prediction, and treatment prognosis [19,20]. However, only integrating this knowledge into neurocomputational models can researchers identify active neural representations and circuits. This approach can guide the development of neurotechnologies that directly target pathways responsible for bionic motor execution [21]. Moreover, human motor performance displays great variability and adaptability when responding to task demands. The central nervous system (CNS) organizes numerous neuromuscular elements into coordinated patterns to meet the complexities of motor output [22]. This demands more refined target settings that integrate biophysical signals into computational models while ensuring that the restoring movements are both mechanism-based and human-like.

Theoretical neuroscience has contributed to uncover the complexities of neuromusculoskeletal systems and make therapeutic decision to simulate natural motor behaviors [23,24]. The motor primitive (MP) model, in particular, has integrated mathematical, neurophysiological, and bioengineering expertise. It can explain how movement repertoires are formed from spatiotemporal combinations of pre-organized units called MPs or synergies [25]. Machine learning algorithms can then decompose natural motor tasks—considering kinematic, muscular, and neural aspects—into sets of motor modules [26]. This helps explain how the CNS coordinates peripheral motor elements, such as muscles and joints, to ensure task efficiency, adaptability, and motor variability [27]. Furthermore, the flexible MP combination can regenerate diverse motor output patterns with high degrees of freedom (DOFs) shared by the human beings despite individual variations. This model can possibly inspire the development of human-like practice/stimulus templates in NGNTs.

For clinical aspects, effective protocols still require optimization to minimize the trial-and-error costs across diverse populations, although neurotechnologies are acceleratingly emerging [8,28]. Following theoretical neuroscience paradigm, we argue that target setting is a pivotal issue in resuming coordinated motor control, serving as a foundation for the development of bionic neurotechnologies. This narrative review begins by discussing conventional target settings used in HMI protocols, including clinical, kinematic, muscular, and neural metrics. We then highlight how the MP model can consolidate multimodal motor representations through machine learning methods and provide neurophysiological insights into neuromotor dysfunctions. Finally, the NGNTs inspired by the MP model are summarized, with a focus on their practical application scenarios. While translating novel neurotechnologies into clinical applications remains challenging, theoretical models can help researchers cross disciplinary boundaries. We believe the MP model sets a promising landscape to inspire different neurotechnology instantiations for restoring human natural movement in the near future.

## Gaps in Target Setting for Guiding Naturalistic HMI

In this review, we define “human natural movement”’ by replicating its inherent biomechanical and neurophysiological traits of humans, aimed at guiding its regeneration in naturalistic HMI. It refers to a continuous, flexible, efficient, and intuitive motor output in the sensorimotor process as natural as possible. Beyond HMI, target setting is a kernel configuration for motor restoration in the field of rehabilitation. It differs from biomarker of intervention response (for example, demographics, diagnosis, and evaluation) for precision medicine to identify target population and predict treatment success [29]. Instead, target setting establishes the “good” standard of an intervention outcome. It ultimately reflects the neuroscientific interpretation of motor dysfunctions that support subsequent interventional neurotechnologies, for example, promoting neural repair for stroke recovery [30].

**Fig. 1.**
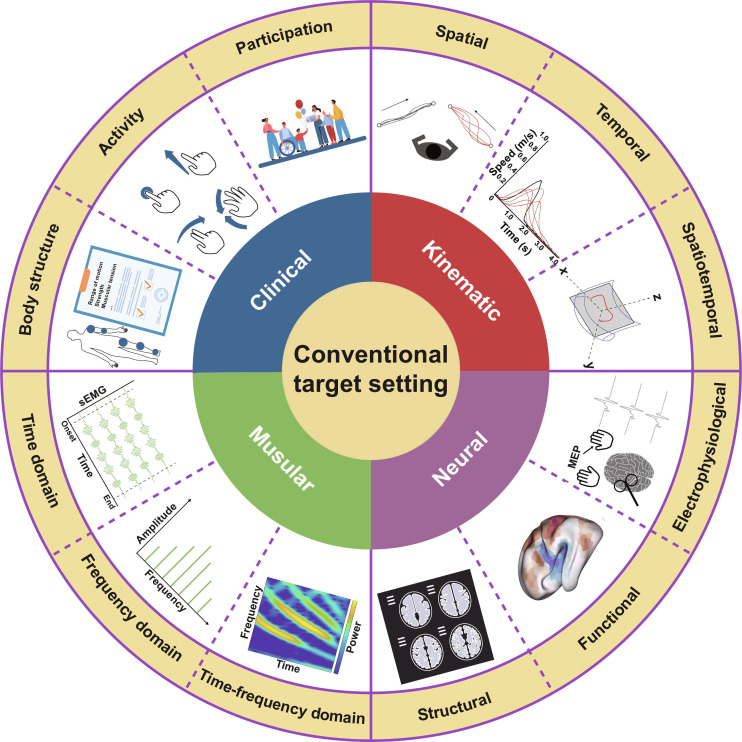
Conventional target setting in restoring motor function, including clinical, kinematic, muscular, and neural aspects.

As mentioned earlier, human motor dysfunctions can be multifactorial and varied (Fig. 1), rendering rehabilitation a complex intervention. Conventional therapy warrants a multitude of actions and presents challenges in designing hybrid systems. To achieve natural movement in the rapid-acting NGNTs, the targets need to represent motor features underlying a neural-mechanistic and information-theoretic model of motor restoration [31]. This target setting aligns with the SMART (specific, measurable, achievable, relevant, and time-bound) principles of regular rehabilitation goals. Here, we argue that the NGNT paradigm imposes higher demands on utilizing kinematic, muscular, or neural metrics by checking against the SMART principle (Box 1) [32].

Box 1. The SMART principle for reshaping target setting in NGNTs [32]

1. S (specific): Clearly specify the biophysical signal(s) to be measured and utilized for HMI in the context of natural motor execution.

2. M (measurable): Include quantitative, sensitive, and objective indicator(s) to measure the progress or success of technological intervention by developing computational models of natural movement.

3. A (achievable): Set alterable metrics that make goals challenging yet realistic to enhance patient’s engagement and performance during human-like practice or stimuli in NGNTs.

4. R (relevant): Tailor the goals to the patient’s specific motor needs, addressing the physiological or biomechanical aspects of motor dysfunctions in comparison to natural conditions.

5. T (time-bound): Establish a clear timeframe for completing each HMI session that simulates natural movements, typically within the range of seconds or even milliseconds.

### Clinical scales

In clinical practice, behavioral scales are commonly used to provide a convenient overview of longitudinal mobility progress following interventions. Many disease-related scales have been developed to manually assess different domains of motor deficits. As per the International Classification of Functioning, Disability and Health (ICF) framework, rehabilitation outcomes can be categorized into 3 levels: body structure, activity, and participation. The ICF framework presents a holistic method for evaluating various aspects of motor function for humans [33].

In the current era of rapid high-technology development, such as artificial intelligence and wearable sensors, traditional scales are facing criticism for relying on subjective ordinal ratings or self-report measures. These limitations have raised concerns regarding observer bias, ceiling or floor effects, and a lack of sensitivity to detect subtle changes during motor recovery [34]. Moreover, clinical scales fail to offer sufficient and timely insights into the biophysical phenomena underpinning motor recovery-information. This is imperative for developing more targeted and individualized neurotechnologies in the HMI process.

### Kinematic metrics

Motor sensors embedded in neurotechnologies can detect biomechanical information during interventions [35]. These data enable the calculation of spatial, temporal, and spatiotemporal metrics, which characterize participants’ motor performance and deviations from desired actions. Spatial metrics encompass variables such as joint angles, distances, and body part displacement. They help understand movement trajectories and accuracy. Temporal metrics analyze movement duration, speed profiles, efficiency, and interjoint timing coordination. Lastly, spatiotemporal metrics provide insights into velocity profiles, acceleration patterns, and smoothness [36].

When employing kinematic parameters to deliver human-in-the-loop practice in NGNTs, it is crucial to recognize the limitations in goal setting against the SMART principle. Kinematics alone cannot directly capture the neural processes that underlie human natural movement. Notably, diverse patterns of neuronal activity make it complicated by encoding and transmitting motor commands. Human motor execution involves the sophisticated recruitment of motor elements regulated by specific neural circuits. Synchronizing these neural ensembles is essential for precise and coordinated output [37]. Furthermore, single or multiple indicators often fail to comprehensively represent motor performance or characterize how “good-enough” it is [38]. Thus, advanced algorithms are necessary for intelligent kinematic interactions in NGNTs to regenerate natural movements.

### Muscular metrics

With the development of flexible electronics, surface, intramuscular, and high-density electromyography (sEMG/iEMG/HD-EMG) have been utilized to study control strategies for neurotechnologies with millisecond precision. Motor execution-related parameters typically include time, frequency, and time–frequency domain features [39]. Besides, advanced algorithms such as deep learning are increasingly utilized in this field [40].

In achieving motor restoration, traditional EMG parameters primarily focus on individual muscle activity or offline classifications of motor intention. However, they tend to oversimply movement naturality in humans. The real-time recruitment and interplay among muscles can be overlooked. This lacks detailed information into the timing and activation patterns of muscle groups during complex motor tasks [41]. Furthermore, these parameters face challenges in distinguishing between maladaptive and compensatory actions. As with kinematics, EMG recording provides indirect measure of the underlying CNS signals responsible for initiating and controlling muscle contractions [42]. Consequently, computational models are warranted to decode neuromotor coordination by accounting for the contributing properties of descending neural pathways (for example, corticospinal, rubrospinal, and reticulospinal tracts).

### Neural metrics

Neurologic examinations have substantially enhanced the understanding of the anatomical and functional integrity of the nervous system (Table 1) [43–45]. Many techniques are available to demonstrate neural hallmarks in persons with mobility dysfunctions. However, the main challenge lies in elucidating the dynamic motor process using indicators of neuronal activity or network representation [46]. Typically, interpretations are anchored indirectly to clinical scales by investigating their statistical associations. Data-driven analyses, like machine learning on large-scale datasets or casual models of neural dynamics, have also facilitated clinical prediction and phenotype classification [47,48]. On temporal-spatial resolution, fusing multiple neural signals allows for a trade-off among examinations, such as integrating functional magnetic resonance imaging (fMRI) with electroencephalography (EEG).

**Table 1.** Overview of common neural techniques for evaluating motor dysfunction

Aspects of assessment	Neural techniques	Principle	Metrics
Structural integrity	Computerized tomography (CT)	Structural visualization of neural tissues	Lesion location and volume
	Magnetic resonance imaging (MRI)		
	Diffusion tensor imaging (DTI)	Structural architecture and connectivity of white matter tracts	Fiber orientation, density, and integrity
Functional imaging	Functional magnetic resonance imaging (fMRI)	Blood oxygen level-dependent (BOLD) signal	Indirectly reflect neural activity and connectivity with higher spatial resolution
	Functional near-infrared spectroscopy (fNIRS)	Oxygenated/deoxygenated hemoglobin concentration	
	Functional ultrasound imaging (fUSI)	Hemodynamic response	
	Positron emission tomography (PET)	Glucose metabolism	
	Electroencephalography (EEG)	Neuronal electrical activity	Directly measure brain activity and connectivity with higher temporal resolution
	Magnetoencephalography (MEG)	Neuronal magnetic activity	
Neurosurgical techniques	Single-unit activity (SUA)	Epidural, subdural, or cortical activity	Directly record neural activities with high temporal and spatial resolution
	Multi-unit activity (MUA)		
	Electrocorticogram (ECoG)		
	Microelectrode arrays (MEAs)		
Neurophysiological technique	Transcranial magnetic stimulation (TMS)	Excitability of corticomotor tract or intracortical neuronal circuits	Motor-evoked potential

Nevertheless, decoding neural activity patterns into motor behaviors via digital models is still a marked area of concern. Cross-validation of spatiotemporal series with behavioral scales may appear less rigorous in explaining coordinated movements. This is especially true given that the scale itself may not be best for depicting behavioral performance. Some neural findings could be also epiphenomena or stochastic noises of coordinated motions. Furthermore, structural findings may be less responsive to immediate NGNT intervention; functional network metrics may not inform the continuous neural population activity. These shortcomings make target setting less attainable during time-bound HMI sessions.

## MP Model: Toward a Unified Framework for Restoring Human Natural Movement

Computational modeling is crucial for quantifying the spatiotemporal characteristics of human natural movement. In this case, the MP model provides a unifying framework to decode the complex neuromuscular coordination required for volitional movements. To be analogous, motion primitives serve as the fundamental building blocks for the skilled tasks, much like how alphabetic characters constitute the gradient foundation of linguistic system [49].

By conceptualizing movements as flexible combinations of basic functional units, this model offers a tractable solution to the challenge of high-dimensional motor control. It integrates interdisciplinary insights from neurophysiology, computational modeling, and machine learning, thereby facilitating the development of NGNTs. Through intelligent algorithms, the MP model enables extraction of latent motor patterns from vast data. This can effectively map motor intentions with interpretable subspaces in humans. Neurophysiological evidence further supports this model. It has demonstrated that MPs are encoded and modulated across hierarchical neural circuits, which enable efficient motor execution, adaptation, and relearning [50]. Taken together, the MP model not only explains the biological basis of motor coordination but also serves as a computational scaffold to exploit the machine intelligence properties of human body. Hereinafter, we will present how the MP model may boost clinical translation of NGNTs with its theoretical neuroscience and experimental findings.

### Conceptual premise

In order to achieve volitional movements, the human CNS must efficiently coordinate the innervation of numerous muscles within its motoneuron hierarchy [51,52]. Managing individual commands for multiple DOFs in parallel could be overwhelming for the generation of diverse motor behaviors. At this point, the MP model conceptually indicates that a few basic motor blocks flexibly form human natural movement. It can robustly delineate multiple representations of functional movements and propose an integrated hypothesis for interdisciplinary crosstalk. Moreover, neural networks are speculated to facilitate the contribution of individual muscles to diverse contexts, thereby building bridges for developing NGNTs aimed at addressing mobility impairments [24].

**Fig. 2.**
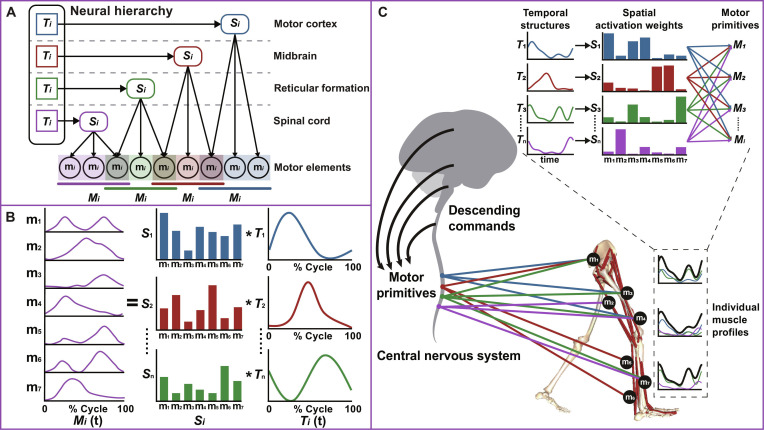
Schematic diagrams of the motor primitive (MP) model from conceptual, mathematical, and neurophysiological views. (A) Conceptual premise of the MP model. The complex neuromuscular properties of human natural movement are organized within the neural hierarchy of the CNS. Motor elements are surrogated by the MPs that are encoded by the motoneuronal systems with their spatiotemporal parameters of *S_i_* and *T_i_*. Adapted from Cheung and Seki [51]. (B) Mathematical schematic of the MP model. By using dimension reduction algorithms, the profiles of human natural movement can be decomposed into temporal structures and the respective spatial activation weights. Adapted from Dominici et al. [200]. (C) Neurophysiological interpretations of the MP model as exemplified in the lower limb. The generation of coordinated motor sequences entails interactions of the motor cortex, midbrain, reticular formation, and spinal neurons for the precise timing and intensity of MP recruitment. Adapted from Ting et al. [53] and Neptune et al. [201].

Different descending tracts project to divergent but interlaced motoneuronal pools that control multiple muscles. Given this, the movement repertoire of human is postulated to be composed of a shared group of basic blocks with respective neural origins. This principle suggests that the complex neuromuscular properties can be effectively surrogated by the MPs. Motor elements *m_i_* are encoded hierarchically by the motoneuronal systems with various primitives *M_i_* with their spatiotemporal parameters of *S_i_* and *T_i_* [53], as depicted in the schematic diagram (Fig. 2A). Meanwhile, MPs can be reshaped by motor experience or training through the fine-tuning of neuronal circuits. This hypothesis explains the phenomenon of motor relearning in rehabilitation therapies. As a result, high-dimensional movements can be biomimetically decoded into subspace of motor intentions that require fewer control signals. Such findings lay the foundation for developing computational modeling and theoretical analysis under the MP model, which further translates human-like properties into machine intelligence of NGNTs.

### Synergy analysis algorithms

Mathematically, human natural movement requires complex neural calculations to orchestrate the precise timing and amplitude of appropriate motor elements. As such, computational models have come to the fore. They enable digital readouts of coordinated neuromotor activities to preliminarily support the human-in-the-loop scheme in NGNTs [54]. To identify MPs, data derived from kinematic, muscular, or neural recordings typically undergo processing with the mathematical idea of dimensionality reduction. This process is known as “synergy analysis” or “synergy extraction”. The algorithms have predominantly encompassed nonnegative matrix factorization (NNMF), principal components analysis (PCA), and artificial neural network (ANN) [55].

Through synergy analysis, the original data matrix is often linearly decomposed into 2 matrices in a concise fashion. To be brief, the motor profiles can be mapped by the synchronous organization of temporal structures *T_i_* and their respective spatial activation weights *S_i_* (Fig. 2B) [56]. This allows ensembles of various motor elements, acting as latent variables, to be spatiotemporally constrained in equations for compatible motor execution across different contexts [57]. Apart from dimensionality reduction, the core mathematical methods of synergy analysis can span to other branches of machine learning. They include deep learning and reinforcement learning (RL), each with its own scope of application. Deep learning involves specific models like convolutional neural networks (CNNs) and recurrent neural networks (RNNs). It should be emphasized that not all applications of such algorithm qualify as synergy analysis. Instead, the algorithms embody considerations of various hierarchical levels in human natural movement control and simplification of DOFs. To emulate building modules like interneurons within neural circuits, computational models incorporate intermediate or latent variables. With this design, the conceptual premise of MP can be realized by algorithms.

### Neurophysiological mechanism

The MP model has also accumulated evidence from neuropathway experiments. Since the CNS needs to manage motor elements to achieve redundant task solutions, the MP model simplifies this motion intelligence issue by assembling them into elementary building patterns. This intricate process entails interactions of the motor cortex, midbrain, reticular formation, and spinal neurons [58]. It has been supported by extensive evidence from intracortical microstimulation in vertebrates, invasive neurostimulation, and causality analysis of focal neural injuries (for review, see Cheung and Seki [51]). With the MP model, neural innervation of human natural movement can be underpinned by the primitive-encoding cortical activity in conjunction with somatosensory afferent fibers. These neural pathways ultimately converged onto motoneuron pools via sensorimotor synapses [59]. Hereby, the CNS can employ intuitive and simplified motor commands. By clustering MPs onto spinal motor neurons, it then invokes elaborate activation level and timing of multiple joints or muscles for desired actions. As exemplified in Fig. 2C, the individual muscle profiles are organized by MPs that are driven by descending commands from the CNS.

**Fig. 3.**
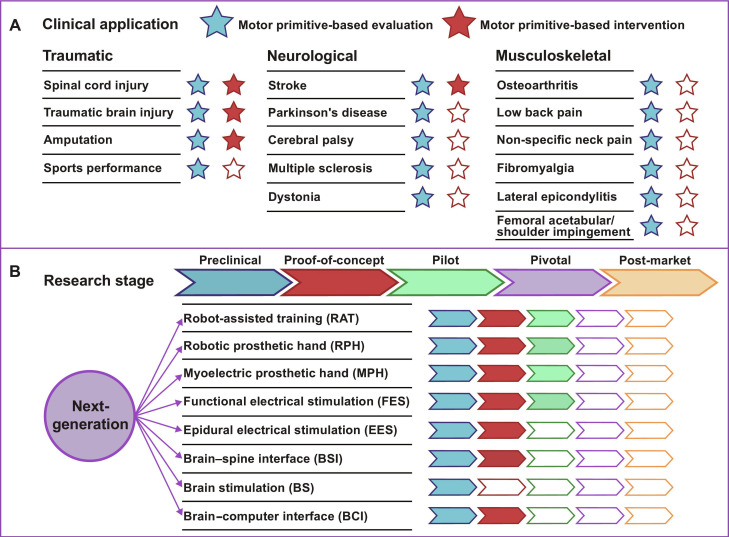
Clinical applications of evaluations and interventions based on the MP model. (A) Involvements of MP model in common traumatic, neurological, and musculoskeletal diseases. (B) Research stage of the next-generation neurotechnologies inspired by the MP model. Clinical trial stages of medical devices generally span from preclinical, proof-of-concept, pilot, pivotal, to post-market trial. Most of the neurotechnologies are at preclinical and proof-of-concept stages.

As the research progressed, the structural basis of the MP model has gradually become clear. Neuroanatomically, the primary motor cortex (M1) plays a crucial role in initiating and executing movements by transmitting fine-grained neural impulses to the peripheral motor units [60]. This process cannot be achieved without the support of other structures within the CNS. Interconnected neural circuits exist across other brain areas, such as dorsal premotor cortex (PMd) and primary visual cortex (V1). These circuits can constitute neural manifolds to explicitly represent the almost simultaneous neural dynamics of specific tasks [14]. With evolving understanding of neural networks, the brainstem, cerebellum, and spinal cord have been identified as relay stations. They transmit signals from the brain cortices to innervate the peripheral motor elements by coding MPs [61]. Furthermore, these structures generate neuronal circuits for reflective modulation of MPs. Notably, the modulation process involves the ventral interneurons that compose central pattern generator (CPG) in the spinal cord [62–64]. Consequently, this model offers an innovative neurophysiological perspective on motor execution and impairment in various conditions (Table 2 and Fig. 3).

**Table 2.** Motor impairment in various conditions from the perspective of motor primitive model

Disease	Motor dysfunction	Pathophysiological interpretation	MP-based explanation
Stroke	Weakness, abnormal muscle tone, decreased coordination, impaired dexterity	Lesions in motor-related brain areas or descending motor tracts caused by cerebrovascular accident	Altered structure and abnormal coupling of motor primitives [180]
Spinal cord injury	Complete/incomplete tetraplegia or paraplegia	Traumatic or nontraumatic damage to the local spinal cord circuits and sensorimotor fibers that transmit high-level neural signals	Lost motor primitives Abnormally structured motor primitives Disrupted recruitment of motor primitives
Parkinson’s disease	Tremor, rigidity, bradykinesia, postural instability	Degradation of dopaminergic neurons in the midbrain basal ganglia	Inappropriate selection of motor primitives
Cerebral palsy	Muscle strength, muscle tone, reflexes, gross/fine motor abnormalities [206]	Damage to or abnormalities inside the developing brain, including periventricular leukomalacia, cerebral dysgenesis, intracranial hemorrhage, and inadequate supply of oxygen [207]	Reduced number of motor primitives Immature development of motor primitives [208]

### Exploitation of machine intelligence

The MP model is not merely a tool for analyzing biological data to learn human natural movement. Instead, it enables emulation of human machine intelligence in NGNTs by actively modeling, simulating, and refining functional outputs to rebuild physiological structures [65]. Specifically, by encoding complex motions algorithmically, the model leverages the CNS’s modular logic to control peripheral effectors. These MP-driven features form the fundamental framework for various NGNTs. Consequently, a key research priority has emerged to replicate the biomechanical and neurophysiological traits of human movement. Novel sensor and chemical materials thus have more applications in such rehabilitation devices. For instance, as will be detailed in following section, kinematics from human hands can be decomposed into MPs and further integrated into prosthetic design to restore dexterity after amputation. Additionally, real-time tactile sensing can be converted to the weighting of these MPs for refining sensorimotor circuits [66].

Apart from supporting engineering fabrication, the MP model translates biological efficiency of human body into natural control. As human natural movement is proven to be intuitive, the MP model achieves this by decoding motor intentions in a way analogous to CNS. It turns abstract neural signals into purposeful, intuitive actions. This replication shows up in generating coordinated movements by adaptive control with sensation inputs [67]. To further align with human-like adaptability, machine intelligence of basic motor blocks is leveraged here. It uses closed-loop feedback to bidirectionally refine movements over time just as the brain rewires itself to improve functionality [68]. For example, adaptive BCIs analyze neural spikes to identify and cluster “primitive-like” motion patterns, then dynamically adjust these patterns to generate movements that feel natural to humans.

## NGNTs for Motor Restoration

Conventional neurotechnologies often rely on direct decoding or device-centric mappings that treat biophysical signals as discrete control channels. This yields brittle, task-specific control struggling to reproduce the coordinated qualities of human movement. The central limitation lies in the mismatch between human–machine targets and the physiological organization of motor control, which is modular and low-dimensional despite high-DOF biomechanics.

In the NGNTs, MPs are spatiotemporal building blocks encoded across hierarchical circuits that CNS flexibly combines to generate diverse behaviors (Table 3). NGNTs use MPs as the mechanistic target and computational scaffold. They extract MPs from kinematic/myoelectric/neural data and then recompose those primitives to drive naturalistic outputs in real time. By aligning device control with this latent modular structure, NGNTs reduce control dimensionality and preserve human-like timing and coordination of motor output.

**Table 3.** Comparison between current-generation and next-generation neurotechnologies

Dimensions	Current-generation neurotechnologies in restoring human natural movement	Next-generation neurotechnologies inspired by the MP model
Core model/algorithm	∙ Rely on practical signal classification such as EMG pattern matching for prosthetics or fixed operational rules ∙ Apply basic control scheme with a focus on function realization rather than deep integration with biological logic ∙ Maintain stable performance for routine tasks but have limited adaptability to dynamic movement demands	∙ Center on the MP model, decompose movement into modular blocks by machine learning such as NNMF and PCA ∙ Employ closed-loop adaptive algorithms to align with neuroplasticity ∙ Fuse multi-modal data including kinematics, EMG, and neural signals for natural movement control
Design principle	∙ Follow engineering-driven design that prioritizes device functionality and ease of use in rehabilitation, including acoustic, photonic, mechanical, electrical, thermal, and magnetic modalities ∙ Adopt standardized protocols that work for most users, reducing complexity in setup and operation for wide-scale deployment	∙ Follow more advanced, biology-driven design, aligning with CNS’s hierarchical motor control spanning cortex, brainstem, and spinal cord ∙ Patient-specific, adjusting modular combinations to individual biomechanics such as tuning MPH based on amputees’ residual muscle activity
Mechanism of action	∙ Achieve assist-as-needed movement such as robotic training for stroke, which helps maintain joint mobility and prevent muscle atrophy ∙ Support neuroplasticity through repeated practice/stimuli, with a focus on functional activation rather than direct mimicry of MP mechanisms	∙ Drive activity-dependent neural plasticity such as RAT with human-like templates to rebuild naturalistic motor patterns ∙ Achieve mechanistic motor restoration by mimicking CNS’s MP-based control, such as modulating spinal circuits via EES
Scope of application	∙ Focus on simple motor output and basic rehabilitation such as hook prosthetics for daily grasping tasks ∙ Widely used in acute rehabilitation such as post-stroke robotic training to lay a foundation for early recovery	∙ Support natural movement such as MP-based RPHs for dexterity, EES for voluntary walking, and RAT for coordinated reaching ∙ Applicable to chronic conditions with even neural plasticity promotion
Hardware	∙ Adopt reliable devices that meet basic rehabilitation needs ∙ Ensure acceptable biocompatibility for regular use ∙ Operate independently across device types, which simplifies setup for single-function applications	∙ Use miniaturized, body-integrated systems such as EES implants and textile-based tactile sensors ∙ Feature modular plug-and-play design, such as BSI integrated with EES for spinal cord injury
Software	∙ Conduct offline signal processing using fixed patterns for standard scenarios ∙ Maintain stable, easy-to-implement operation for consistent tasks but cannot dynamically adapt to motor variability such as changes caused by muscle fatigue	∙ Realize real-time MP decomposition and tuning motor synergies ∙ Apply closed-loop control such as ECoG decoding in BSI systems ∙ Provide sensory feedback, such as tendon vibration to enhance embodiment
Advantages	∙ Technologically mature, low-cost, and easy to access such as basic myoelectric prosthetics that benefit large user groups ∙ Simple to operate and deploy in clinical settings such as standard FES for stroke rehabilitation, supporting efficient routine care	∙ Generate continuous, flexible, efficient, and intuitive motor output ∙ Highly adaptable, adjusting to task demands such as RPHs for fragile objects ∙ Potentially high generality, working for several diseases by tuning MPs
Disadvantages	∙ Generate movement that meets exercise needs but is less natural such as robotic motion in standard training modes ∙ May see reduced efficacy over long-term use ∙ More suitable for simple tasks such as basic grasping, with limited support for complex multi-joint movements	∙ Have high development costs and risks such as invasive BSI requiring surgical implantation ∙ In early clinical stage with small sample sizes ∙ Require complex calibration, needing personalized MP extraction via machine learning

In addition to characterizing the CNS how to deal with motor coordination, the MP model has inspired the revolution of neurotechnologies, despite most of which are at preliminary stages of clinical trial. By driving or sculpting various MP aspects, these approaches can be generalized to restore coordinated movements for large populations with traumatic and neurological insults. Firstly, harnessing the flexible combination of kinematic primitives makes it possible to exploit the biomechanical limb characteristics of healthy populations for task-specific control. This capacity facilitates robotic human-like movements, which helps reshapes motor behaviors in neurological diseases. It also enables high-level reconstruction in robotic prosthetic hands (RPHs) for individuals with limb amputations. Secondly, myoelectric activities provide templates for 2 key applications: peripheral electrical stimulation and biomimetic control of neuroprosthetics. Emulating physiological muscular primitives, these devices may enhance neuromuscular control in persons with neuromotor impairments. Lastly, mapping functional tasks to neural primitives supports the development of novel neurostimulation techniques and BCIs. It is achieved by mimicking the way the CNS coordinates human natural movement. These techniques involve the targeted modulation or activation of neural circuits to either promote specific motor functions or alleviate motor deficits. As such, they hold promise for improving functionality and life quality with the biomimetic solutions soon (Fig. 3).

### Kinematic level

#### Next-generation robot-assisted training

Robot-assisted training (RAT) has been widely used to administer repetitive and interactive task-specific training for upper limb rehabilitation following stroke [69]. Its development, as an electromechanical approach, was predominantly driven by engineering idea for more than 30 years. Moreover, conventional RAT is grounded in a theoretical basis of motor relearning to maximize neuroplasticity, mainly including brain reorganization, interhemispheric balance, and neural compensation [70]. Consequently, training modalities (i.e., passive, assisted-as-needed, active, resistive, and error-augmentation modes) and control strategies have been the focal points within the robotic community [71,72]. Reconsidering the principles of activity-dependent neural plasticity, motor recovery could be optimized by functional tasks and patterns that are directly trained [73]. However, current-generation RAT paradigms largely involve planar or mechanized motions, which are distinct from coordinated patterns seen in human natural movement [74].

**Fig. 4.**
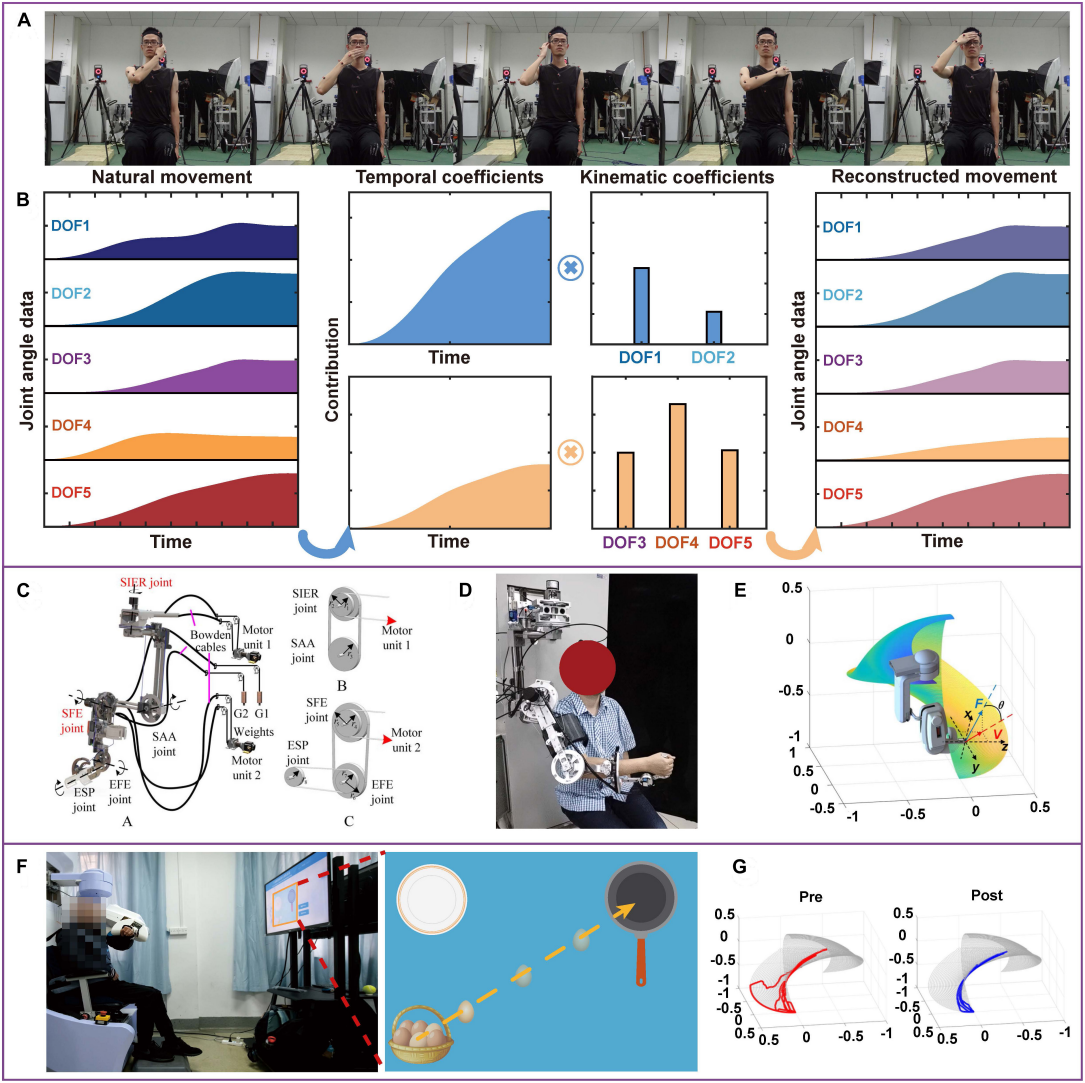
Next-generation robot-assisted training (RAT). (A) Joint angle data of several upper limb activities in healthy adult were recorded using optical motion capture. This information was exploited for reproducing human-like movement. (B) Dimensionality reduction algorithm was used to extract postural information in the form of kinematic primitives. (C to E) Mechanical and control configuration of the synergy-based RAT. (F) Human-like RAT program was used for post-stroke rehabilitation. The participant received physiological movement training under the guidance of exoskeleton. (G) Smoother endpoint trajectory in workspace of the kinematic reaching task was shown after intervention. Figures adapted from He et al. [76] and Chen et al. [78].

One may consider integrate human-like kinematic features into the RAT regimens. Based on the MP model, Chen et al. [75] reproduced upper limb postural profiles by exploiting joint angle information of healthy adults. They employed optical motion capture for several arm movements, such as hand-to-mouth/ear tasks (Fig. 4). Kinematic synergy analyses via PCA and cluster analysis were mainly involved in their paradigm to regenerate shoulder, elbow, forearm, and wrist motions with 5 active DOFs and 2 passive DOFs. Consequently, several postural primitives were extracted to reconstruct human-like training references through the Jacobian matrix. This information was also robotized to constitute anthropomorphic motions and interaction manifold in the exoskeleton by accounting for more than 80% of motor variances [76,77]. In a randomized controlled trial, *N* = 80 patients with subacute stroke were included. They were found to diminish postural error and recover smoothness after repetitive training with robotic human-like movements. Notably, robot-assisted anthropomorphic movement training may help restore arm motor function and activities of daily living for the participants, with improvements of 14.73 points in Fugl-Meyer Assessment for Upper Extremity [78]. We believe that this technology presents a robotic template by emulating human natural movement via a combination of kinematic primitives. It holds the potential to boost motor recovery and mitigate learned misuse after stroke [79].

#### Next-generation RPH

Artificial devices have made progress in restoring motor function for individuals with amputation. Nonetheless, conventional body-powered prostheses frequently suffer from poor user experience, including unaesthetic appearance, limited range of motion or comfort, and inhuman-like movements [80,81]. Among veterans with unilateral upper-limb loss, those who rely on no prosthesis or only cosmetic device report markedly worse activity limitations and perceived disability (*β* = 9.4; *P* = 0.0004). Technically speaking, it is challenging to replicate the motion intelligence of human hand functionality. Continuous control of prostheses is also difficult when classifying the operator’s online intentions through the exhaustive enumeration of all possible finger motions derived from large training datasets [82]. Given to these reasons, the attempt to replicate human hand structure with a bionic system would directly support seamless control.

**Fig. 5.**
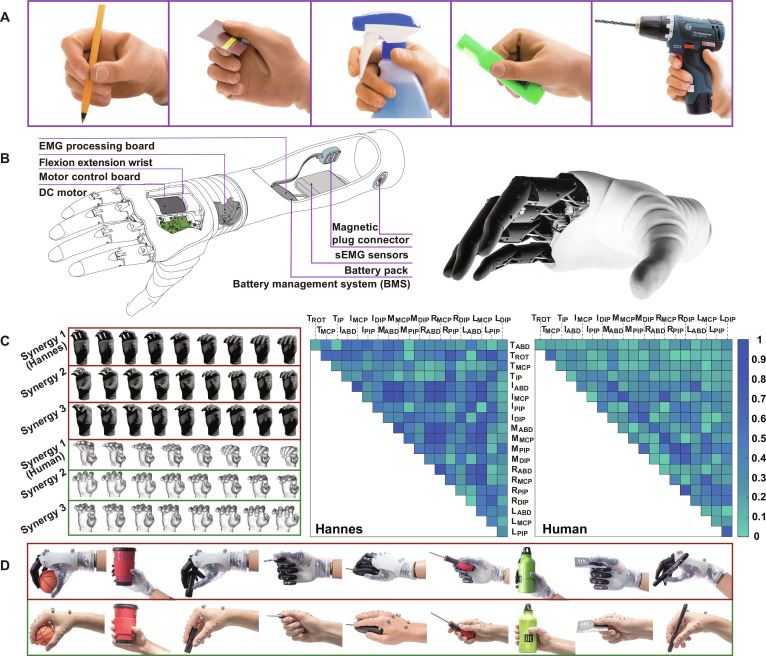
Next-generation robotic prosthetic hand (RPH). (A) Daily grasping activities that were replicated as combinations of synergistic motion patterns. (B) Poly-articulated RPH that can generate anthropomorphic hand patterns. (C) Comparison and correlation of the principal MPs by robotic and human hand. (D) Grasping postures by the RPH hand and its resemblance to natural human hand movements. Figures adapted from Laffranchi et al. [65] and Naceri et al. [202].

In recent years, the RPH has witnessed substantial progress in mechanical engineering, material science, and artificial intelligence to realize prosthetic embodiment [83]. By targeting kinematic synergy of daily activities, it becomes possible to prospectively characterize motor properties of the human hand. A key challenge in achieving natural actions with robust control is developing bio-inspired systems. Such machine intelligence contents are conducive to intrinsically execute the postural primitives of high-DOF motions, such as grasping different objects looking like humans. Exemplified by the Hannes RPH, it replicates a wide range of high-performance grasping capabilities by allowing patients to directly control 2 to 4 kinematic primitives (Fig. 5) [65]. Using PCA, 97% of hand variance can be identified with 3 PCs. Further similarity analysis demonstrated high humanoid degree in the Hannes hand. As indicated in the feasibility study, the synergy-based approach is highly significant for regaining human-like dexterity, including grasping bottle, computer mouse, and screwdriver. Through a 2-week program, *N* = 3 participants relearned how to operate the synergistic mechanical configuration. These advancements suggest that the next-generation anthropomorphic RPH offers individuals greater functional independence in household settings, thus seizing more possibilities in the real world [84].

### Myoelectric level

#### Next-generation myoelectric prosthetic hand

Apart from the anthropomorphic performance indicated in RPH, another challenge is to achieve natural myoelectric control with limited physiological information after hand amputation [85]. As illustrated in mathematics, treating each of the hand’s *N* muscles as simply on or off yields 2*^N^* possible activations. Generally, the multi-articulating mechanical systems are preprogrammed and proportionally controlled, instead of being independently driven with discrete control commands [86]. Myoelectric time series in the residual arm are then processed to interpret intended movements of the patients, nonspecific to the task phases. Despite advancements in detecting motion intentions, there can be issues with accurate, real-time, and biomimetic control properties through the myoelectric interface. The common solution is to make a trade-off between myoelectric prosthetic hand (MPH) dexterity and control flexibility. Surgical procedure to create muscle–machine interface, such as targeted muscle reinnervation (TMR), can be an alternative approach for restoring functional control [87].

**Fig. 6.**
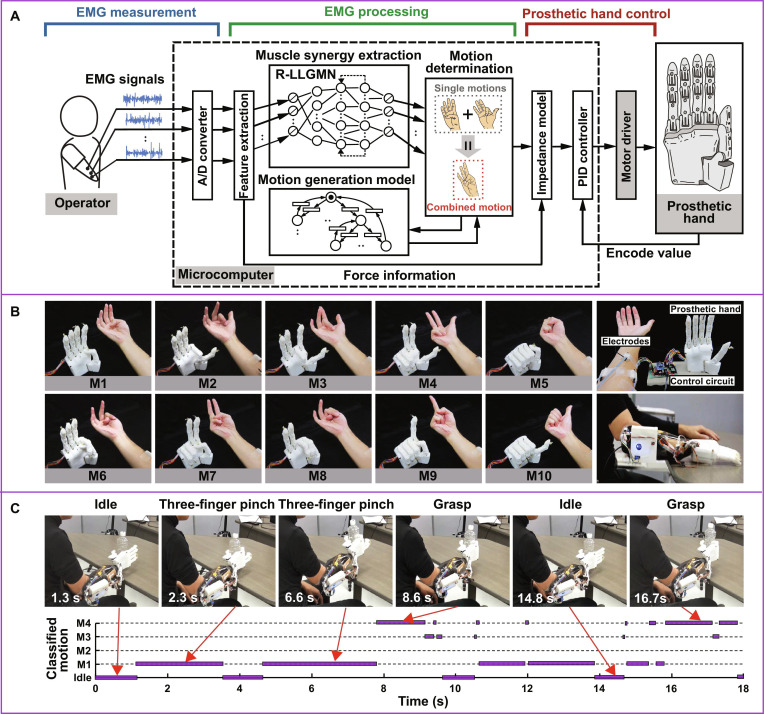
Next-generation myoelectric prosthetic hand (MPH). (A) Muscle synergies were extracted from human natural multichannel EMG signals, which were transformed as prosthetic single motions (M1 to M5) and then recombined into functional motions (M6 to M10) as shown in (B). (C) The MPH showed high accuracy of recognizing motion task and phase in actual scenes for the individual with hand amputation. Figures adapted from Furui et al. [88].

Impressively, Furui et al. [88] proposed a noninvasive MPH that integrates muscle synergy-based motion determination and impedance model-based biomimetic control (Fig. 6). The system utilizes the MP model to determine fundamental finger motions from the residual sEMG signals with a microcomputer. In this study, the individual finger motions were embodied in the MPH by reliably matching the extracted muscle synergies throughout the motor period. To be specific, log-linearized Gaussian mixture network was applied to identify each single motion with EMG patterns. An event-driven model was further used to predict motions with force information from previous muscle synergies instantaneously. This motion-generation model enabled accurate transition of unlearned functional motions that assemble the learned single motions with >90% classification rate. Moreover, biomimetic control based on an impedance model was employed to maneuver the prosthetic hand, enabling smooth prosthetic movements like human hand movements captured by EMG signals. When picking up plastic bottle from table, the amputee participant could use a 3-finger pinch motion with 2.3 s. Moreover, the MPH switched from idle to 3-finger pinch and grasp naturally by recomposing the basic 10 motions. In short, the synergy-based system could be used for intuitive, adaptive control and improved functionality in an artificial hand. This MP-inspired MPH may ultimately make the life-improving prosthetics more accessible to a larger population with limb loss [89].

#### Next-generation functional electrical stimulation

Functional electrical stimulation (FES) is a portable, transcutaneous neurostimulation technique in motor rehabilitation. It can activate under-recruited muscles in functional movements after neurological diseases, such as stroke. However, the full potential of FES has been underdeveloped due to the lack of a computational approach to refine the pattern of stimulation protocols [90]. Traditional FES paradigms have not quantified the precise timing and intensity of natural muscle contractions in humans. Such protocol makes it difficult to deliver desired stimulation schemes for motor-related cortical plasticity and transforming lives [91].

**Fig. 7.**
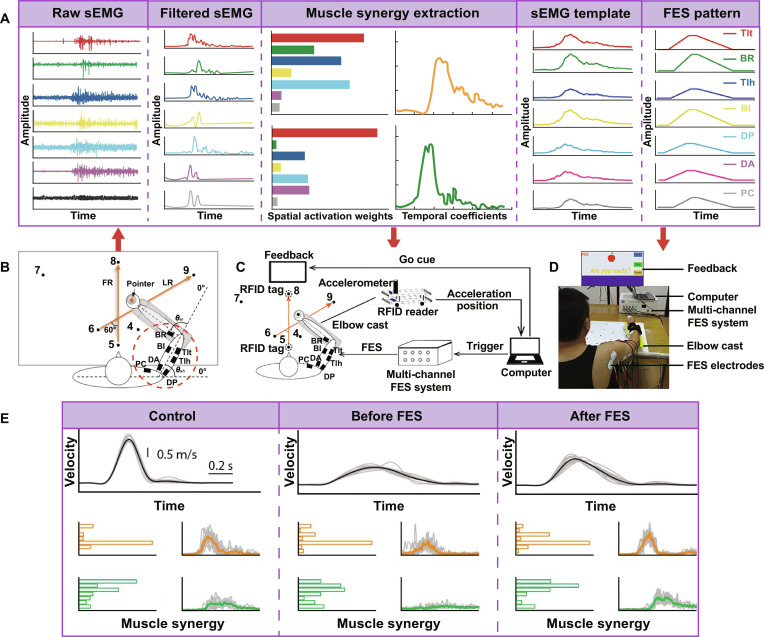
Next-generation functional electrical stimulation (FES). (A) Muscle synergy analysis of human natural myoelectric signals was used to personalize FES patterns. (B to D) Synergy-based stimulation was paired with planar tasks and virtual feedback. (E) Upper limb kinematic performance improved after the MP-based FES intervention. It was associated with the much-normalized muscle synergy patterns as compared with healthy control. Figures adapted from Niu et al. [92] and Chou et al. [203].

Since the MP model may guide the generation of FES patterns, Niu et al. [92] developed multichannel sEMG template during customized arm tasks from a healthy individual in a pilot study (Fig. 7). Muscular primitives were extracted from 7-DOF raw sEMG for reconstituting the natural activation patterns of forward and lateral reaching tasks with the NNMF technique. After the 5-d intervention, the participants showed a mean 13.7% improvements in Fugl-Meyer Assessment for Upper Extremity. Myoelectric patterns also suggested much similarity with the natural template. As a result, the synergy-based FES system was associated with upper limb motor recovery in *N* = 6 stroke patients, standing for a reformative FES paradigm to regulate neuromuscular coordination. In addition, proprioceptive afferent synapses could have innervated the premotor interneurons to encode the recruitment of MPs [93]. As suggested by Cheung et al. [94], MP-based FES may induce rich proprioceptive and somatosensory inputs via afferent pathways to guide activity-dependent neuroplasticity, and then correct abnormal muscle contraction patterns for the executed tasks. We believe that muscle synergy could act as a guiding principle across a wide range of movement repertoires in the next step, including domestic and recreational hand tasks. Technically speaking, it is also expected to enable brain-controlled stimulation through neural decoding, thereby further accelerating stroke rehabilitation. In summary, this approach holds great potential for recovering modular coordination and deserves further investigations in high-quality randomized controlled trials [95].

### Neural level

#### Next-generation epidural electrical stimulation

SCI can lead to refractory paralysis below the level of injury because it disrupts the signals between supraspinal centers and spinal circuits. Biocompatible epidural electrical stimulation (EES) can directly modulate residual neural circuits after SCI. It has shown promise in improving function by activating proprioceptive feedback pathways and increase alpha motor neuron excitability. A review of 21 clinical studies concludes that lumbosacral EES restores sensorimotor function by acting on dorsal root proprioceptive afferents, interneuron connections, and spinal/supraspinal network in humans [96]. However, conventional EES protocols fall short in programming neuromodulation of the spatially distributed motoneurons. They also lack precise temporal sequences to evoke physiological muscle activations in humans [97,98].

**Fig. 8.**
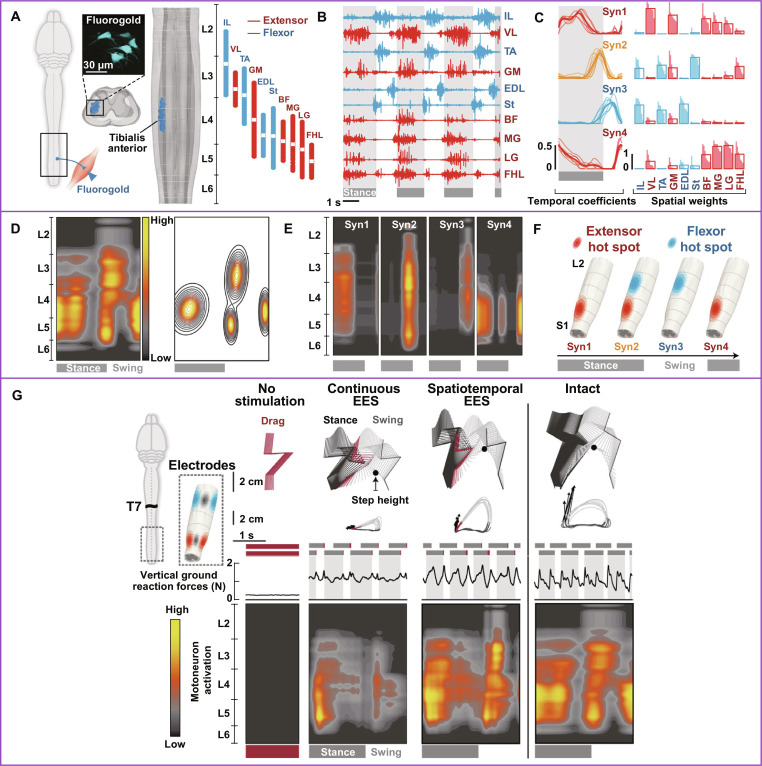
Next-generation epidural electrical stimulation (EES). (A) Rodent hindlimb motoneurons were precisely located for providing the MP-based EES. (B and C) Muscle activity and activation profiles of muscle synergies during locomotion of the intact rat. (D and E) Motoneuron location matrices projected from muscle activity and activation profiles of muscle synergies of the intact rat. (F) Schematic of spatiotemporal EES of muscle synergies with the corresponding hot spots. (G) After complete T7 SCI, rats were implanted with a spinal device. The treadmill locomotion was tested without stimulation, with continuous lumbar-sacral midline neuromodulation, and spatiotemporal neuromodulation. Both hindlimbs’ stance (dark gray), drag (dark red), and swing (light gray) phases and stepping-related vertical ground reaction forces were recorded. Horizontal bars (blue, red, black) indicate electrode states; spatiotemporal motoneuron activation maps used 10 consecutive steps. IL, iliopsoas; VL, vastus lateralis; TA, tibialis anterior; GM, gluteus medius; EDL, extensor digitorum longus; St, semitendinosus; BF, biceps femoris; MG, gastrocnemius medialis; LG, gastrocnemius lateralis; FHL, flexor hallucis longus. Figures adapted from Wenger et al. [99].

Next-generation EES shares similarities with muscle synergy analysis using NNMF. By decoding 4 muscle synergy profiles during quadrupedal locomotion in healthy rats, Wenger et al. [99] identified the optimal electrode configurations (2 hot spots in the lumbosacral spinal cord). The muscle synergies consisted of both extensors and flexors. Four MP profiles that weighted combinations of the 10-DOF muscle activity sufficed to reconstruct over 92% of original signal variance. They further used Gaussian cluster algorithms to recruit proprioceptive feedback circuits corresponding to stance, stance-to-swing transition, and late swing (Fig. 8). Moreover, the coordinated sEMG signals, which match the natural dynamics of motoneuron activation, were regenerated through real-time software adjustments of the implanted electrode. Continuous EES delivered to L2–L3 and S1 midline segments enabled all rats tested to achieve coordinated treadmill-induced locomotion. The results demonstrate that synergy-based EES significantly improve gait quality, weight-bearing capacity, and skilled locomotion. Although this proof-of-concept study primarily focuses on rodent model (*N* = 6), the novel scheme presents a next-generation EES. Remarkably, it explored the neuronal subpopulation responsible for regaining walking function after SCI in humans (see Kathe et al. [100] and Wagner et al. [101]). This protocol was further extended to restore arm and hand function (*P* < 0.05) in a preliminary trial of 5 patients with cervical SCI [102]. Following these translational medicine studies, Cheng et al. [103] further proposed the rShiftNMF algorithm, which improved classification accuracy by over 11%. They mapped muscle activity to spinal cord segments and applied the biomimetic EES technique to a small case series (*N* = 2) with complete SCI. The results offer valuable insights into residual muscle synergies, as the neurocomputational infrastructure, to support potential brain-actuated or paired EES application for restoring motor function after SCI [104].

#### Next-generation brain–spine interface

**Fig. 9.**
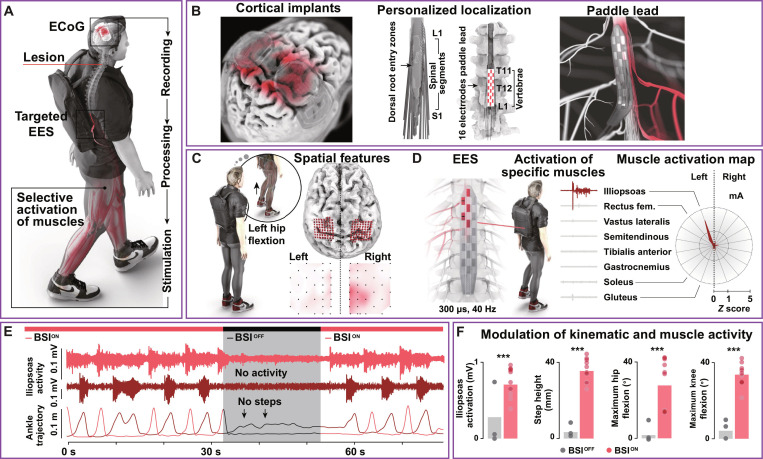
Next-generation brain–spine interface (BSI). (A) The BSI was configured by electrocorticographic (ECoG)-triggered EES that bypassed the spinal lesion in human. (B to D) Here, ECoG signals were identified to initiate synergy-based EES for reproducing natural walking function for individual with spinal cord injury. (E) Iliopsoas activity and ankle trajectory with and without BSI. (F) Changes in iliopsoas activation, step height, and maximum hip/knee flexion degree before and after BSI. Figures adapted from Lorach et al. [107].

In harnessing the power of synergy-based EES on the lumbosacral spinal cord, the above research team further unlocked a new realm of bypassing the lesion for restoring brain-controlled walking function after SCI [105]. Apart from targeting the locomotor muscle synergies, brain-derived commands can initiate the preprogrammed stimulation profiles. With intact electrocorticographic (ECoG) signals, the sustained effects of stimulation are important aspects for volitional control outside the laboratory [106]. To bridge the functional communication between the brain and spinal cord, the synergy-based brain–spine interface (BSI) consists of the implanted recording, wearable processing unit, and stimulation systems (Fig. 9) [107].

Likewise, the dorsal root neuronal populations were treated with the established EES. It reproduced physiological muscle synergies for standing and walking in a person with chronic SCI. As a significant stride forward, the BSI incorporated ECoG recording of the sensorimotor cortex to enable robust, wireless, and real-time volitional MP recruitment. Weighted Markov-switching multilinear model was used to decode the probability of lower limb intentions from soft mixing of expert predictions. This advancement fosters more adaptive control by ECoG-reflected brain dynamics to bypass the SCI lesion. It allows individuals to seamlessly fine-tune specific timing and amplitude of neurostimulation with their motor intentions with personalized surgical locations. The BSI enables continuous intuitive walking control with strong robustness. Spectrogram and amplitude modulation also demonstrate robust performance during voluntary pauses. Remarkably, even when the BSI was closed, the participant retained walking function with crutches in community settings. Gait analysis demonstrated that the patients had 72% improvements in Time up and go test (*N* = 6), showcasing the enduring neurological impact of this restorative technology. This proof-of-concept study shows that the BSI facilitates immediate lower limb movements. In addition, it may promote neuroplasticity via reorganization of neuronal pathways in the long run. Overall, the BSI represents a monodirectional digital bridge driven by movement-related neural representations. This MP-inspired NGNT holds the potential to revolutionize neurorehabilitation for motor deficits in broader populations [106].

#### Next-generation brain stimulation

Brain stimulation (BS) techniques can modulate the neuronal transmembrane potentials of intracortical circuits and enhance neural plasticity to achieve functional recovery [108,109]. These therapeutics have been mature in motor rehabilitation. The most common forms of noninvasive BS are transcranial magnetic stimulation (TMS) and transcranial direct/alternating current stimulation (tDCS/tACS) [110]. Notably, premotor neuron typically innervates the motoneuronal pools of divergent muscles. Consistent with this, previous studies have shown that the BS-evoked local electric field can activate a spatiotemporal set of muscles for probing the neural basis of MPs [111,112]. However, conventional BS paradigms still fail to generate dedicated brain state or multi-muscle activation analog to those of human natural movement.

**Fig. 10.**
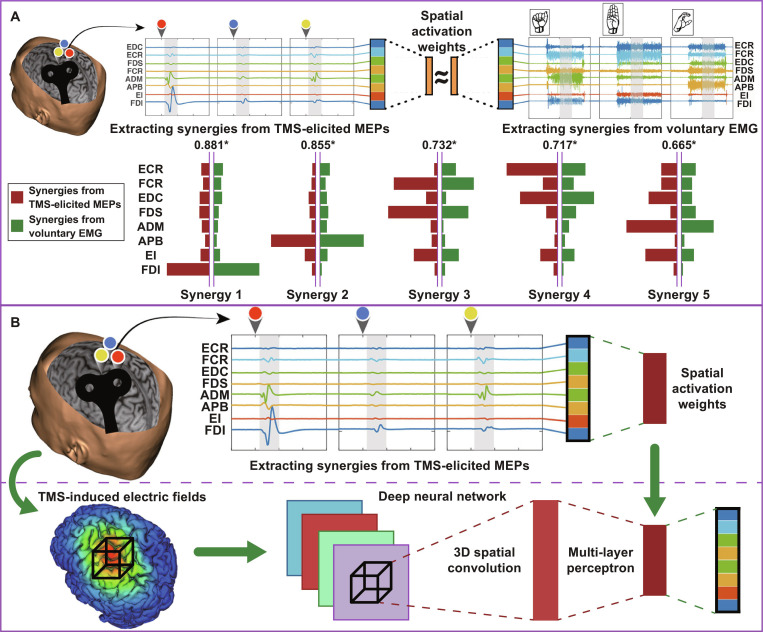
Next-generation brain stimulation (BS). (A) Transcranial magnetic stimulation (TMS) to the motor cortex elicited hand–forearm muscle synergy profiles similar to those of voluntary hand movements. (B) TMS-induced electric fields in the motor cortex were used to simulate the MP-based BS paradigm for functional muscle activation with a deep neural network model. ECR, extensor carpi radialis; FCR, flexor carpi radialis; EDC, extensor digitorum; FDS, flexor digitorum superficialis; ADM, adductor digiti minimi; APB, abductor pollicis brevis; EI, extensor indicus; FDI, first dorsal interosseus. Figures adapted from Yarossi et al. [113,204].

Inspired by the MP model, motor-evoked potentials in the targeted muscles can be extracted with the NNMF technique. Previous study has indicated that the 8-DOF spatial activation weights are similar in TMS-elicited and voluntary conditions. These MPs may in turn harness the noninvasive BS to elicit natural motor patterns intrinsic within the neurons [113]. A major challenge is reliably locating the brain motor cortex that maps to the desired muscle grouping. By computationally modeling neural stimulation to the evoked muscle synergy, Akbar et al. [114] proposed a deep CNN called M2M-Net (motor cortex to muscle network). The pipeline of 3-layer mapper can induce coordinated activation in 15 hand–arm muscles during the refined TMS (Fig. 10). Using the neural response profile as input, different architectures and fitting schemes were explored to develop the model. As a result, selective muscle responses can be reproduced across various TMS settings, including coil configurations, pulse characterization, and stimulus intensity. This synergy-based therapeutic framework could potentially enlighten the field of high-precision BS by producing normal network signatures, including deep BS and transcranial electrical stimulation [115,116]. Moreover, using CNN architectures offers advantages when BS profiles and neuromuscular responses, as well as their causal relationship, include numerous parameters. Yet, further verification of the safety, feasibility, and clinical effects of coordinated muscle activation is necessary for its future implementation.

#### Next-generation BCI

In the field of BCI, extensive research has focused on converting neural signals via motor imagery-based classification [117]. The goal of this conversion is to trigger/augment the external devices (for example, exoskeleton, electrical stimulation, and prosthetics) in traumatic or neurological conditions [118,119]. Due to the remarkable complexity of human movement, restoring motor actions often requires compromises. A common one is to reduce the bandwidth of neural commands or simplify the DOFs of task output [120,121]. As a plain attempt, Benabid et al. [122] focused on synthesizing given functional tasks. To do this, they preprogrammed the natural activation profiles of the targeted muscles or robotic architectures. In the famous BrainGate2 trial, this approach has been demonstrated to be viable by Ajiboye et al. [123] Interestingly, it was used to build a 36-channel, intention-triggered, and predefined intramuscular FES that was paired with the intracortical BCI (FES + iBCI). In this proof-of-concept demonstration, a tetraplegic participant regained coordinated reaching and grasping movements, for example, retrieving a cup of coffee and drinking using a straw.

**Fig. 11.**
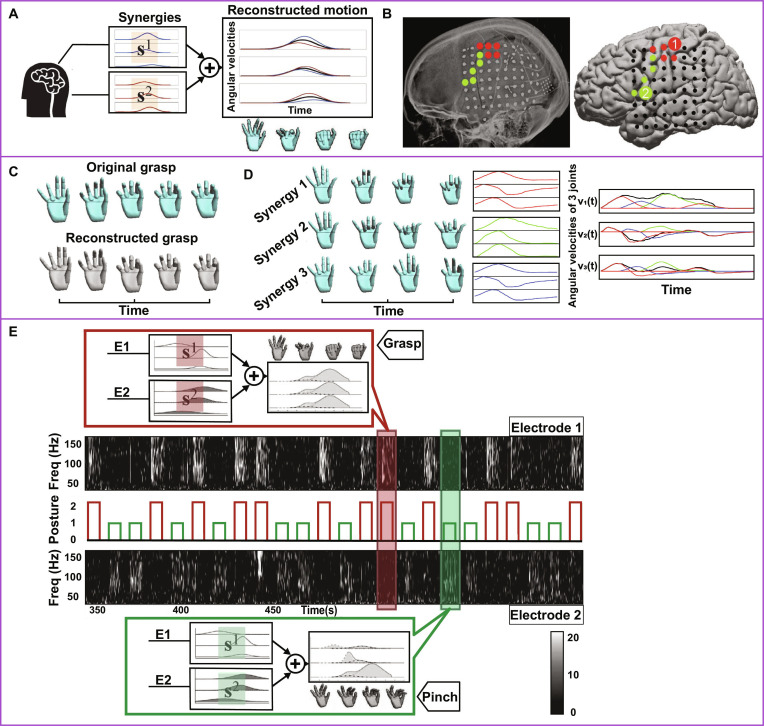
Next-generation brain–computer interface (BCI). (A and B) ECoG signals from 2 electrodes were recorded to map the neural primitives of hand movements. (C and D) Kinematic synergies resemble joint-angular-velocity profiles for reconstructing grasping movements in the BCI. (E) Cortical activity of the synergy-based BCI was used to control the high dimensional virtual hand. Figures adapted from Vinjamuri et al. [124,205].

To support continuous control with ECoG signals, Vinjamuri et al. [124] presented a synergy-based BCI. They preestablished the control of high-dimensional virtual hand postures using low-dimensional intracranial command signals (Fig. 11). MPs of finger joints during daily activities were extracted with convolutive-mixture model in 5 individuals. Two synergies were identified to map a 10-DOF virtual hand, namely, the 2-finger pinch and whole-hand grasp. The synchronous and asynchronous synergies showed high similarity index of 0.90 to 0.99. ECoG signals were further calculated as the average spectral power in the high gamma band (75 to 115 Hz). These signals were recorded from 2 electrodes implanted in the parietal, temporal, and posterior frontal lobes. The characteristic ECoG patterns of neural synergy were demonstrated to be feasible for real-time control of the dexterous prosthetic hand in the participant with intractable epilepsy. Compared with noninvasive method (like EEG), invasive BCI can increase the signal-to-noise ratio of brain commands [125]. Nonetheless, only 2 neural activation patterns were decoded from the relatively large brain areas and 64-disc electrodes for mapping motor outputs. We believe that artificial intelligent algorithms interpreting mapping results can aid in determining optimal electrode positioning and fabrication. Microelectrode arrays and transcranial neuromodulation could be the potential alternatives to acquire or manipulate more accessible channels for synergy-based BCI control [126,127]. Additionally, despite that Gaussian filter-based synergies simplify the subject’s transformation from task to synergy space, the present algorithm may limit the number and accuracy of hand postures that can be achieved. Therefore, further research and development of synergy-based BCIs are still required to provide simultaneous and multidimensional control of natural movements in neurological conditions.

## Discussion

### Why MP-inspired neuroethologies may represent the next-generation therapeutics for restoring human natural movement?

Distinguished from current rehabilitation approaches, the MP model provides unifying principles that allow crosstalk of neurotechnologies for motor restoration. We propose that NGNTs are not simply incremental engineering advances, but rather innovations inspired by neural evidence and computational infrastructure of human natural movement. Instead of focusing only on signal process or device configuration, NGNTs embed modular control, neural manifold representation, and predictive coding into their designs [128]. This theoretical framework guides the interpretation of natural signals and the optimization of HMI, allowing NGNTs to transcend the limitations of current technologies (Fig. 3) [129].

These features can be articulated in several key dimensions. First, NGNTs achieve closer mechanistic proximity to the neural manifolds of motor control. They are designed to align with the latent structures of neural dynamics that naturally govern movements. By targeting the modular premise with digital modeling, they regenerate more naturalistic and intuitive control than approaches that rely on a fixed manner [130]. Second, NGNTs demonstrate superior spatiotemporal precision to make motor outputs human-like. Interventions are spatially localized and dynamically adapted to motor intents. Third, safety is enhanced by adopting less invasive interventions because human–machine interfaces can be accurately localized. Besides, adaptive algorithms are employed to adjust task demands and regenerate functionality of human body. Fourth, the DOF scalability of controllability allows these systems to move beyond “the curse of dimensionality” [131]. Binary or single-joint actuation is progressed toward multi-joint synergistic control, thereby restoring movements that approximate the richness of human motor repertoire. Finally, we envision that their less-redundant structural designs reflect advances in understanding the machine intelligence of human anatomy. Integrated with existing rehabilitation workflows, it may lower the threshold for translation rehabilitation device into clinical practice [132].

At the kinematic level, although anthropomorphic robot and prosthesis sit furthest from CNS, they are the most mature and scalable up to date. These approaches, along with synergy analyses, capitalize on human-like kinematic primitives to shape behaviors and reduce compensatory patterns [133]. The strengths show immediate deployment for motor reconstruction via prostheses or hybrid systems like BCI. From an engineering perspective, robotic arm requires just 2 independent synergies that explain at least 80% of interlimb coordination variance in hemiplegia [134]. However, at the same time, kinematics falls indirect with respect to the true generators of movement. Robot optimizes motor patterns without necessarily engaging the underlying neural representations. It may ultimately cap long-term generalization and neuroplastic change after neural injuries.

Myoelectric-level approaches (synergy-based MPH and FES) occupy a middle ground. These techniques exploit muscular primitives with millisecond resolution, providing greater physiological specificity than kinematics while avoiding risks of neurosurgical access [135]. They enable intuitive control when the EMG structure is at least partially preserved, and deliver biomimetic activations to reestablish modular coordination [136]. Similar to myoelectric assessment, several constraints persist. Electrode instability, muscle fatigue, and distinguishing adaptive from maladaptive synergies in real time are inevitable. In patients with severe paresis or amputation, usable EMG may be sparse for intuitive control schemes [85].

Neural-level approaches (synergy-tuned EES, BSI, BCI, and BS) are closest to the putative substrates of MPs and best positioned to drive targeted plasticity in humans. They offer the highest temporal precision for volitional continuous control with neural oscillations. More importantly, restorative effects for natural movement by decoding neural signals can be further amplified through external devices. However, they distribute across a spectrum of invasiveness, from noninvasive BS to fully implanted BSI, each with relevant risks, costs, and maintenance demands [137]. As mentioned earlier, neural-level NGNTs have shown striking capability in well-selected case series and controlled settings. But their generalization to daily use remains challenging due to calibration drift, hardware fragility, and limited bandwidth for complex human behaviors [138].

In short, kinematic-, myoelectric-, and neural-level NGNTs are not mutually exclusive competitors but rather complementary levers along a mechanistic gradient for naturally restoring human movements [26]. The most promising path forward is layered integration: kinematic templates to structure practice or stimuli, myoelectric interfaces to enforce peripheral modularity, and neural interventions to engage or reconstitute central motor representations, each framed within the MP model to keep target setting mechanistic and human-like. Meanwhile, alternative computational or conceptual models could be considered to complement the MP framework for better guiding motor restoration in various diseases.

### Alternative motor control models

While the MP model provides an interesting scaffold for hierarchical composition, recent frameworks of motor control could enrich NGNT designs. Uncontrolled manifold (UCM) offers perspectives of motor variability. It emphasizes redundancy management and contributes to stability while allowing flexibility in movements. Variability confined to dimensions that leave the performance variable unchanged can be tolerated or even exploited [139]. Whereas equilibrium-point control focuses on impedance regulation, advocating that variability along task-relevant dimensions should be minimized [140]. It complements the MP model by providing explicit guidance on how variability can be structured during movements [141]. These methods inform current design of assist-as-needed strategies and impedance-based prosthetic controllers. Aspects of flexible motor outputs are similarly highlighted in these models without compromising task performance. However, they remain largely descriptive and lack detailed neural implementation, limiting the ability to fully capture underlying neural mechanisms that the MP model seeks to represent [142].

Optimal feedback control theory (OFCT) hypothesizes that CNS combines predictive forward models with state estimation to minimize task-relevant costs under perturbations. Multiple effectors of human natural movement emerge from optimization for task goals rather than fixed modules [143]. Given this, NGNTs can embed cost-aware controllers and adaptive estimators that co-learn task objectives with users [144]. OFCT complements the MP model by explaining how CNS dynamically integrates predictions and sensory feedback to optimize motor commands reacting to environmental stimuli [145]. This model primarily focuses on optimality principles and task-level performance, but offers limited insight into the basic modules that directly drive coordinated muscle activation.

Dynamical systems emphasize low-DOF neural manifolds and transient dynamics within motor cortex and its connected networks. Latent variables capture smooth, time-varying population trajectories that govern movement (for review, see Wang et al. [146]). It has been indicated that parametric representations fall short of capturing intricate structure of population-level activity during reaching [147]. These approaches can complement the MP framework by elucidating how coordinated motor outputs can arise from neural dynamics. Their implications for NGNTs include the need for decoders and stimulators to align with latent manifold geometry along desired neural flow fields. Nevertheless, due to being abstract away from discriminative muscle-level control, guidance for translating latent neural dynamics into high-DOF motor actions remains limited.

Active inference and RL address motor behaviors under uncertainty as probabilistic inference or policy optimization. These frameworks provide mechanisms for reward-driven personalization and co-adaptation, which are crucial for cognitive control in NGNTs [148]. Hierarchical RL naturally dovetails with MPs that sub-policies can serve as modular building blocks composed into larger skills, enabling efficient learning and transfer. In this way, RL and active inference complement the MP model by embedding mechanisms for ongoing adaptation, while MPs ground these policies in neurophysiological plausibility [149]. However, both frameworks mainly operate at abstract policy, offering less direct insight into how motor modules are naturally assembled in real time.

Taken together, these alternatives are not opponents to the MP model for innovating neurotechnologies. Instead, we believe that a hybrid and flexible program is attractive [150]. MPs supply interpretable physiologically anchored building blocks across muscle/kinematic periphery, while dynamical systems and manifold models can guide alignment with neural population activities. UCM/equilibrium-point theories regulate variability and impedance in outputting motor programs of control schemes. Moreover, others provide normative principles for adaptive control and exploitation. Embedding NGNTs with such frameworks may yield modular controllers and stimuli that are both clinically explainable and performance-optimal.

Whether using MP or another modeling approach, digital protocols are required to simulate neuromusculoskeletal manifestations to disentangle black box of motor coordination in humans. Thankfully, a hybrid of diverse approaches involves advanced biosensors and artificial intelligence algorithms. It is increasingly contributing to decoding biophysical signals for motor restoration in technologies. We believe that more standardized experimental pipelines and computational procedures may create practical NGNT solutions for transforming lives.

### Sensory feedback as implicit component in MP model

Before taking motor behavior as modular, reafferent feedback is well-acknowledged to shape natural movements [150]. Within the sensorimotor loop, proprioceptive, tactile, vestibular, and audiovisual cues cooperate over multiple timescales from estimating current state to predicting future states. As mentioned in its neurophysiological basis, fast spinal/brainstem loops stabilize dynamics for the MP model; cortical loops update motor plans and parameters; and reward-based mechanisms consolidate new mappings. Consequently, adding appropriate sensory channels is an implicit but prerequisite for motor regeneration in NGNTs [151]. In protocols such as RAT or BCI, enriched feedbacks can potentially gate plasticity and align practice with desired neural representations. In RPH or MPH, well-timed tactile, proprioceptive, or spinal information can reduce cognitive load and improve user performance. For example, Sagastegui Alva and colleagues [152] successfully integrated spinal reflex pathways into the HMI process. They found that RPH control can be improved naturally through closed-loop tendon vibration, and the participants with amputation saved more than 12 s in the Box and block test.

Given the modular characteristics, conventional efference copy cannot be used directly for HMI in NGNTs [153]. Here, we argue that MP framework indicates a principled adaption of sensory input at the motoneuron level [154]. Feedback can be delivered from peripheral (cutaneous tactile, vibrotactile, skin stretch, tendon vibration), neural (dorsal root and intracortical microstimulation), or mechanical (torque and impedance cues) levels [155]. However, feedforward channels in NGNTs are suggested to reorganize task-relevant commands at the level of MPs [156,157], for example, grasp aperture and contact state for a whole-hand grasp, joint timing, and force pulses for reaching, or phase-specific cues for locomotor synergies. This alignment reduces controller complexity, preventing mismatches that may cause sensory conflict in HMI schemes [158].

Recent advances indicate that 2 design constraints are critical for sensory reorganization. The first is time latency or insufficient information of somatosensory interfaces. It can destabilize closed loops and degrade perceptual quality, leading to maladaptive strategies [159]. This inherent process can be contaminated by noise or the types of sensation, as patients with neural conditions often have concurrent visual or proprioceptive impairments [160]. Electronics can support sensation by eliciting programmable spatiotemporal activity in corresponding neurons [161]. Insights are also gained from neuromorphic electronics driven by neural events. For example, human skin is rich in mechanoreceptors—key for haptic feedback of biomedical systems. By targeting response profiles of such specialized cells, Flavin et al. [162] developed a miniaturized electromechanical structure to enable self-sensing deformation modes. The transducers require only 58 mJ for transitions to generate linear displacements exceeding 2 mm and forces up to 1.4 N. It allows processing input from smartphone-based 3-dimensional scanning and inertial sensors. The second is sensory recoding: Its intensity, frequency, and spatial patterns should respect user experience. As CNS transforms sensory signals into motor perception, feedbacks can lead to counterintuitive estimation of mental workspace in humans. Adaptive feedback scheduling that provides more guidance when learning new synergies and less once they are stable can help internalize movements and avoid over-reliance on external cues [163]. Safety domains should also avoid overstimulation that risks neuropathic pain or desensitization in NGNTs like FES or EES.

It should be noted that feedback serves as a therapeutic mechanism, not merely signal input for engineering regeneration of human natural movement. Sensory signals integrated from neural pathways like natural synergies may help restore neural circuits such as tuning CPGs and promote rewiring of descending pathways [164]. For instance, Parkinson’s disease arises from the loss of dopamine-producing neurons. Departing from general strategies, Milekovic et al. [165] showed that MP-inspired EES of lumbosacral cord can instead be governed by real-time decoding of motor-cortical events in a patient. Moreover, multisensory congruence has potential to enhance body ownership and reduce phantom pain in amputees using virtual reality and electrical stimulation [166]. Some may argue that the MP model underestimates the importance of sensory inputs, which are critical to feedback control and prosthetic embodiment in biomimetic movements [167]. Actually, afferent sensations with external perturbations are intrinsically embedded in the given tasks. They are accompanied with expression of MPs through neural coupling, adaption, or modulation [141]. Bionic control of hand is intuitive. However, interestingly, arbitrary control is more superior than biomimetic control when task difficulty increases [83]. Thereby, the research priority is to quantify how various feedbacks reshape neural manifolds and MP structure over weeks to months, and to personalize feedback to individuals’ sensory thresholds and attentional bandwidth in various task contexts.

### Urgent need for integrating next-generation biosensors

Delivering naturalistic bidirectional HMI to restore human natural movement hinges on sensing technologies. Generally speaking, NGNTs would benefit from sensor suites capable of capturing high-fidelity signals while offering biocompatibility and stretchability. One of the research focus lies in biomimicry: leveraging the evolutionary advantages of natural sensory mechanisms [168]. In biology-inspired electronics field, it aims to detect and interpret diverse stimuli from the environment, later converted into human-interpretable electrical signals for supporting functional interfaces [169]. Ideally, these sensors should achieve precise synchronization across channels during human activities. For example, in prothesis, Iberite et al. [170] used a noninvasive wearable device to deliver thermal stimuli to residual limb skin regions. Participants detected 97.2% of warm/hot (40 °C) and cold (15 °C) stimuli, enhancing embodiment and life quality of *N* = 27 hand amputees.

Apart from biomimetic impetus, how to engineer them into sensory feedback framework matters [171]. In brief, sensor signals should not only seek to replicate user experience but also be exploited as input to properly drive neurotechnologies. To better detect motor intents, multi-node inertial measurement units and strain sensors match optical laboratory fidelity [172]. They can be paired with rigorous calibration, drift compensation, and per-segment alignment [173]. Here, we present a prime example of bioinspired sensors in textile-based wearable gloves. It embeds piezoresistive sensors and vibrotactile actuators into textiles via digital embroidery to reproduce tactile information. Paired with user-adaptive pipeline, it alleviates tactile occlusion and guiding skill learning with 88.6 ± 2.62% haptic feedback accuracy [174]. Moreover, force or torque transducers that are better embedded can quantify interaction with environment. In a recent study, Zhou et al. [175] presented a triboelectric array that achieved 45.1 mV/kPa sensitivity among 40 to 200 kPa to support 94.23% accuracy of gait analysis. For aspects of electrophysiology, flexible electrodes should stabilize signals under sweat, motion, and long wear times. New modalities such as tendon vibration sensing can indicate motor intent when superficial EMG is unreliable. Biocompatibility and wireless monitoring capability are also important for in vivo detection of neural signals. Notably, Tang and colleagues [176] developed a stimulus-responsive, bioresorbable, wireless 8-mm^3^ cubic hydrogel for ultrasonic intracranial monitoring. Implanted via a needle, it deforms with physiological changes to shift ultrasound frequencies, which decouples intracranial pressure, temperature, pH, and flow (10-cm depth) and nearly full degradation in 18 weeks.

At the hardware level, the development trajectory of next-generation biosensors need not strictly adhere to the MP model. Edge computing on low-power microcontrollers or neural processing units supports feature extraction and inference while keeping closed-loop latencies within tens of milliseconds. Battery, thermal, and weight constraints are also spaces to preserve wearability [177]. However, open programming interfaces can help MP-based prostheses, stimulators, and BCIs interoperate. This will support more application scenarios for NGNTs as shown in BSI. In these cases, robust artifact rejection along with adaptive baselining will be necessary to maintain long-term stability [178]. Human factors are accounts to be considered. General priorities include easy donning/doffing, skin tolerance, and minimal daily calibration [173]. For instance, in-socket pressure and shear sensor arrays (plus intraneural interfaces when clinically indicated) can enhance both intent decoding and feedback delivery in RPH or MPH for amputations.

### Considerations of the algorithms for synergy extraction

Machine learning is the algorithmic cornerstone of MP model. Here, we will not introduce the mathematical principles of synergy analysis method in detail, but rather discuss its fusing development with NGNTs. Likewise, neuroscience knowledge has empowered the next generation of artificial intelligence for advanced calculation and decision [179]. We follow a progression of increasing adaptability to complex biological scenarios for replicating human natural movement. Initially, dimensionality reduction methods (e.g., PCA and NNMF) were employed for understanding motor behavior or dysfunctions [27,180]. They transform high-DOF kinematic or muscular data into lower-dimensional space while retaining key information. This provides efficient inputs for modular reconstruction of human-like templates in the aforementioned RAT, RPH, and FES [22]. However, the linear or semi-linear methods primarily focus on simplifying data structures. The inherent probabilistic characteristics in natural environment are hardly modeled, limiting adaptability to dynamic tasks in humans, such as variability in muscle activations.

To address such limitation, more advanced approaches leveraging probability distributions are developed as exemplified in MPH and EES. They characterize biodata patterns at the myoelectric level. By applying Gaussian mixture network or Gaussian clustering, the probabilistic distribution of muscle profiles in human movements can be identified and remodeled. The inherent capacity to handle data uncertainty enables more flexible control. It overcomes the “fixed modules” concerns in traditional dimensionality reduction for synergy extraction [181].

Some may argue that probabilistic methods have limitations. The temporal continuity and state-dependent transitions of biological motion represent an important engineering priority. That is, the switch between different action phases in humans should be captured to reform natural contexts [182]. To fill this gap, prediction algorithms enhance neuromuscular property. Hidden Markov models for state transition modeling or multilinear regression models were integrated for capturing multi-dimensional features. This has supported smooth motor state switching while recruiting MPs in next-generation BSI technologies for a human.

Building with artificial intelligence, deep learning further elevates performance specially at the neural level. It addresses limitations in handling neural network parameters and task-specific intervention variables [183]. For example, convolutional network with its variants excels at adaptively learning complex parameters, including weights and biases in deep architectures. This helps fine-grained spatial features of neuromotor control in models [184]. Critical variables like force control parameters are also fine-tuned for BCI or BS. This capability stems from their unique ability to capture high-dimensional nonlinear relationships, which is hard to model with probabilistic or dimensionality reduction methods.

To facilitate naturalistic HMI, ground-truth labeling of natural movements remains a major bottleneck. Notably, without intelligent algorithms, NGNTs remain limited to controlled environment for sustained function. Scalable solutions include deep learning, self-supervised pretraining on large unlabeled data streams, and micro-assessment embedded in tasks. Algorithmic advances in these areas are winning human’s imagination. As Iskandar et al. [185] demonstrated, tactile sensation does not necessarily originate from tactile sensors. Instead, it was remapped using manifold learning techniques and ANNs to enable intuitive robotic control. Innovations in algorithm further transform traditional HMI paradigms. It strengthens embodiment of therapeutics and help NGNTs mitigate limitations in other domains. For instance, improved signal-to-noise ratio can reduce invasiveness of BCIs, thereby lowering their medical risks. In a recent study, a BCI system incorporated a “copilot” module empowered by CNNs and ReFIT-like Kalman filters. This module infers task goals and assists in action execution, boosting goal acquisition speed by up to 4.3 times [186].

### Potential limitations

Several limitations can temper the promise of MP-inspired neurotechnologies. Conceptually, debates persist about the ontological status of motor synergies/primitives. Some may argue that they are fixed neural modules, emergent solutions to task constraints, or analysis artifacts from dimensionality reduction. Methodologically, synergy extraction is sensitive to algorithm choice, parameterization, and data quality [51]. Real-time decomposition of MPs remains challenging due to nonstationarity and segmentation of overlapping actions. Importantly, other deep learning models (subject to machine learning) are increasingly applied in motor neural decoding [187]. These techniques could greatly challenge the conceptual premise and neurophysiological evidence of MP models, as they do not treat the 2 components as necessary conditions.

Biomedical engineering and clinical limitations are also prominent for this emerging field. Anthropomorphic templates may not transfer to individuals with altered limb biomechanics or atypical strategies. Under these circumstances, aggressive shaping with NGNT risk suppressing beneficial compensations appeared in the patients [188]. At the myoelectric level, monitor is prone to electrode shift, sweat, and fatigue. Invasive systems face surgical risks, hardware longevity issues, and explant considerations, whereas noninvasive systems often lack spatial precision and require calibration that raises maintenance concerns [107]. For clinical trials, many involve highly selected cases with intensive engineering support and short follow-up as shown in Fig. 3. Blinding is difficult, and accompanying treatments can have inflated early gains. As previously described, heterogeneous outcomes and limited head-to-head comparisons obscure effect sizes and clinical generalizability of NGNTs [8]. Lack of pivotal trial along with cost-effectiveness evaluation can limit their administrative approvals. Finally, ethical issues apply equally to NGNTs, including data privacy, cybersecurity, and informed consent for adaptive systems [189].

### Future directions

Advancing MP-inspired NGNTs from novel prototypes to clinical practice requires progress in multiple domains. Scientifically, we recommend developing multi-scale models to link neural dynamics, spinal circuitry, and body biomechanics for restoring human natural movement. Standardized frameworks of synergy analysis are also advocated. These include creating longitudinal datasets that combine high-fidelity sensing data, intervention records, and clinical outcomes to enhance research reproducibility. Additionally, shared evaluation tasks and metrics should be developed to assess the adaptability and control efficiency of these technologies. We further emphasize promoting data sharing to accelerate collective progress in this field [190].

Algorithmically, controllers that support co-adaptation are favored to allow user and NGNT learn from each other. RL estimators can update policies online under safety constraints such as control barrier functions. Universal decoders can map a small set of intent signals to many MPs, and rapid personalization through Bayesian optimization reduces calibration efforts [191]. Closed-loop practice or stimuli should be goal-conditioned with selecting spatiotemporal patterns. As presented in this review, latent neural states should be delivered toward desired regions while accounting for individual motor functionality.

On the hardware side, fully implantable or body-integrated devices can maintain robust biocompatible interfaces. As recent studies show, interoperability standards are necessary to enable prostheses, BCIs, EES, and BSIs to work as modular, plug-and-play ecosystems in the human body [192]. Notably, materials and chemistry will underpin these advances. For biosensors, nanomaterials like carbon nanotubes and quantum dots are game-changers [193]. Their high surface area-to-volume ratios and tunable electrical/optical properties directly boost sensitivity in detecting subtle biological signals. For stimulators and other implantable devices, chemistry-driven solutions offer unique advantages. Hydrophilic polymer coatings, for instance, improve biocompatibility by reducing tissue rejection and minimizing peri-implant scarring [194]. Precision techniques like atomic layer deposition have made ultrathin components for further miniaturization.

Clinically, pragmatic trials are needed with stratified enrollment and outcomes that emphasize participation and life quality. Healthcare systems also need to adapt for recognizing the value of these cyborg and bionic technologies. Ethical frameworks for adaptive devices that modulate autonomy should address consent renewal, transparency, and cyber-safety [195]. Looking ahead, the most impactful NGNTs would likely be hybrid, which combine kinematic structuring, myoelectric enforcement, or neural engagement. All these contents are coordinated by interpretable MP models and normative controllers such as OFCT and RL. Realizing this needs close collaboration across neuroengineering, rehabilitation, and policy, so natural movement can be restored from NGNTs to human daily life.

## Conclusions

This narrative review has highlighted the NGNTs inspired by the MP model for restoring human natural movement. The development of effective paradigms heavily depends on the mechanistic understanding of the neuromotor function or dysfunction underlying coordinated movements in humans [196]. To enhance the effects of NGNTs, a key issue is to optimize target setting during the HMI process. In this view, mathematical constraints constitute the digital infrastructure on reproducing human-like movements. By modeling the human brain and body, it can allow interdisciplinary collaborations from neuroscience, biomedical engineering, artificial intelligence, and clinical efforts. As a theoretical neuroscience paradigm [197], the MP model provides interpretive and computational tools to characterize the biophysical (i.e., kinematic, muscular, and neural) properties of motor representations in functional tasks [198]. Using simple building blocks (MPs), it has translated the hope of digitally reconstructing physiological motor output into nature, exemplified by the synergy-based RAT, RPH, BSI, and more. We believe that this train of thought importantly serves to filter impractical ideas prior to clinical investigations of clinical neurotherapeutics.

Overall, theoretical neuroscience advances of the MP model are inspiring developments in NGNTs and vice versa. By leveraging multidisciplinary advantages, it can promote translational medical research in decomposing and regenerating multi-representation, activity-dependent movements [199]. Apart from this, applications of the NGNTs are informing the biological properties of brain circuits and human natural movement. Ultimately, it helps refine model algorithms, hardware, and software technologies over the long term. However, most of the NGNT systems mentioned above are still in the proof-of-concept stage, with relatively small sample sizes. Further pivotal trials are needed to clarify their clinical importance and pave the way for possible approvals. Despite harnessing innovative technologies with the closed-loop setting, it should be noted that the NGNTs seem not one-size-fits-all approaches considering the heterogeneous populations. Therefore, more clinical studies are supposed to go back informing the optimal intervening protocols (for example, dose, intensity, and MP levels) and patient subgroups to translate these promising techniques into evidence-based practice.
